# Supplementary material for: Understanding the Light-Driven Enhancement of CO2 Hydrogenation over Ru/TiO2 Catalysts
Source: Molecules. 2025 Jun 13;30(12):2577. doi: 10.3390/molecules30122577 (PMC12196449; doi:10.3390/molecules30122577)
Supplement: Supplementary file 1 [file molecules-30-02577-s001.zip › molecules-3580652-supplementary.pdf]

Supporting Information

Understanding the Light-Driven Enhancement of CO_2 Hydrogenation over Ru/TiO_2 Catalysts

Yibin Bu [1], Kasper Wenderich [1], Nathália Tavares Costa [1], Kees-Jan C. J. Weststrate [2], Annemarie Huijser [1] and Guido Mul [1,*]

[1] Photocatalytic Synthesis Group, Faculty of Science and Technology, MESA+ Institute for Nanotechnology, University of Twente, P.O. Box 217, 7500 AE Enschede, The Netherlands; y.bu@utwente.nl (Y.B.); k.wenderich@utwente.nl (K.W.); n.tavarescosta@utwente.nl (N.T.C.); j.m.huijser@utwente.nl (A.H.)

[2] SynCat@DIFFER, Syngaschem BV, De Zaale 20, 5612 AJ Eindhoven, The Netherlands; keesjan@innovencio.nl

* Correspondence: g.mul@utwente.nl

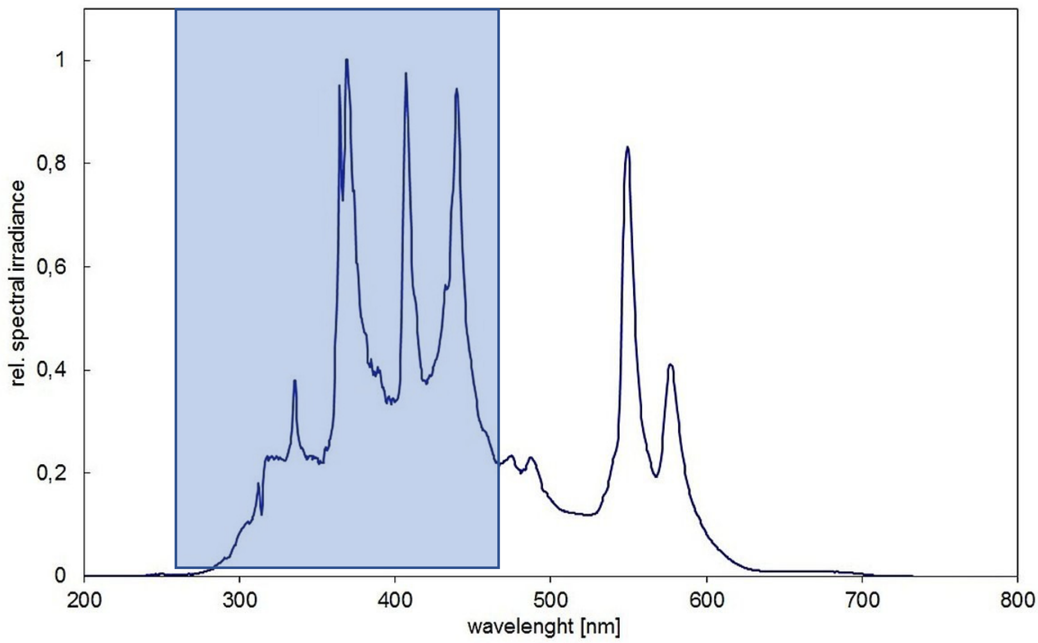

Figure. S1. Emission spectrum of the Hg lamp used in this work for irradiation. The effect of the applied cut-off filter is also illustrated, removing the emission intensities below 420 nm. The illumination was introduced to the sample cell, via a fiber optic cable, causing significant losses of intensity.

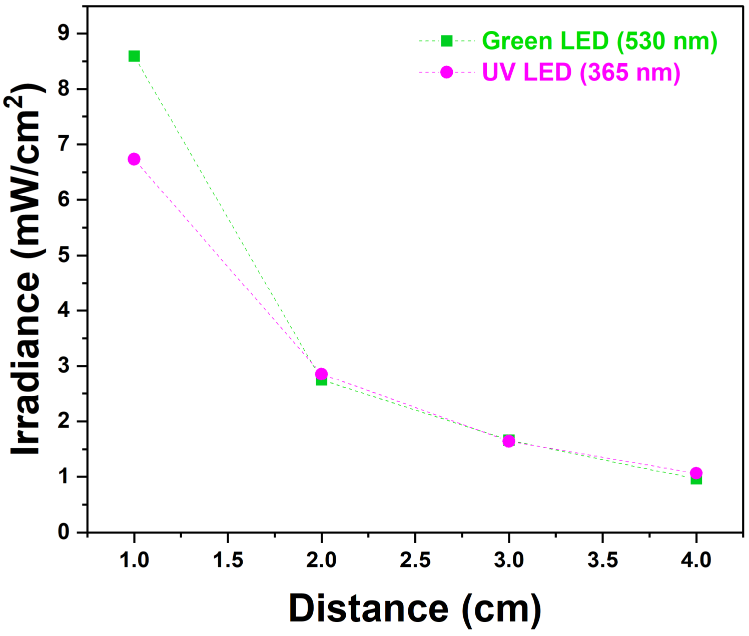

Figure. S2. Intensity vs distance curves for the two LED light sources used in this study. The distance between the light source and the sample amounted to ~1 cm, where the irradiance of the UV LED source amounts to ~ 7 mW/cm^2, and of the green LED amounts to ~8.5 mW/cm^2.

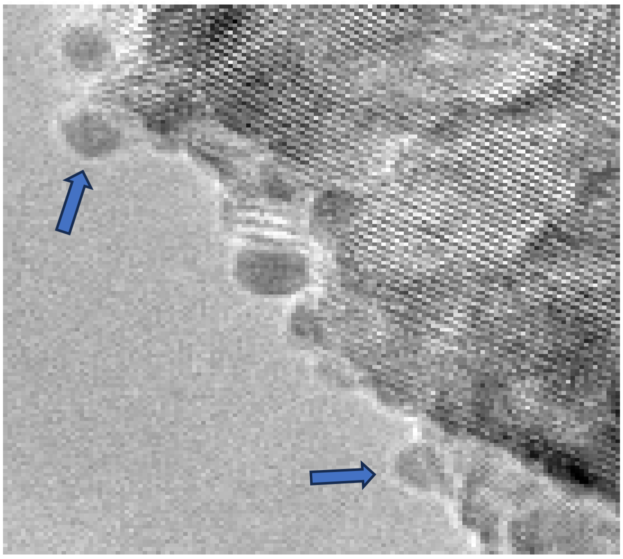

Figure. S3 Enlargement of the TEM image of the Ru/TiO_2 particles, obtained after reduction in H_2 flow. Clearly the amorphous overlayer is visible in these samples, and the morphology of the particles similar to previously reported in the literature[29,30]. Higher resolution images are required to substantiate the nature of the overlayer, and exclude imaging artifacts (e.g. Fresnel fringes along the particle edge).

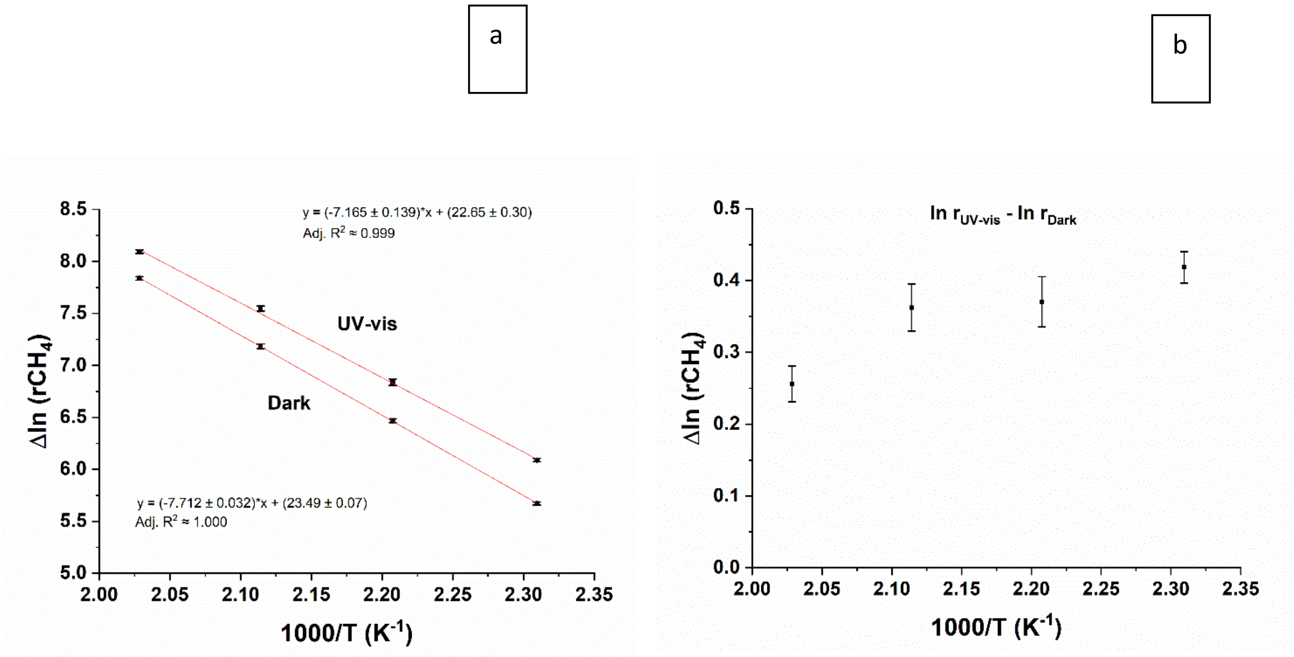

Figure. S4. (a). Arrhenius plots of CH_4 formation on Ru/TiO_2 in dark and under UV-vis irradiation. From the slope of the curve (equal to 1000* E_a/R) and the intersection with the y-axis (equal to ln(A)), we calculate in the dark E_a = 64.1±0.3 kJ mol^{-1} and A = (15.9±1.1)*10^9 s^{-1}, while under UV-vis illumination we find E_a = 59.6±1.2 kJ mol^{-1} and A = (6.9±2.1)*10^9 s^{-1} (1st order kinetics is assumed). Although minor differences are found, we believe that the differences are too small to justify that it originates from a change in reaction mechanism. (b). Comparison of the difference in ln r as a function of 1/T. Considering the figures of the main text (shifting by 10 °C resulting in large overlap) we anticipate the difference is relatively constant as a function of temperature.

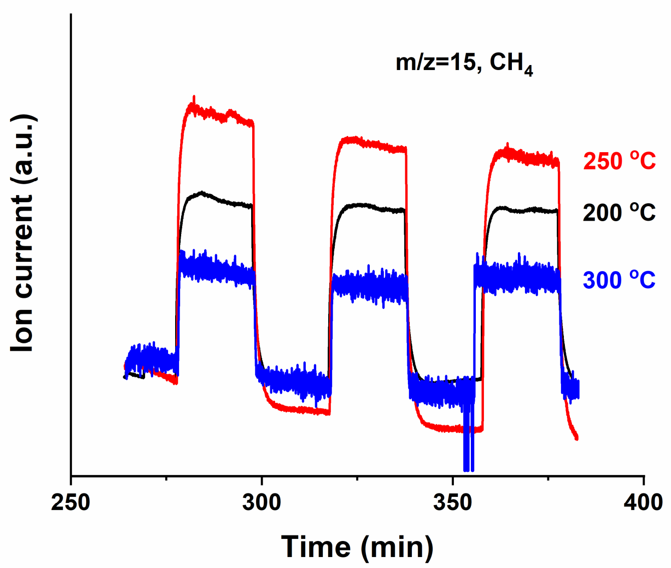

Figure. S5. Zoom in of the CH_4 response of the Mass Spectrometer in photothermal conversion of CO_2 and H_2 in dark, and under UV-vis irradiation as a function of temperature, the largest response is obtained at 250 °C.

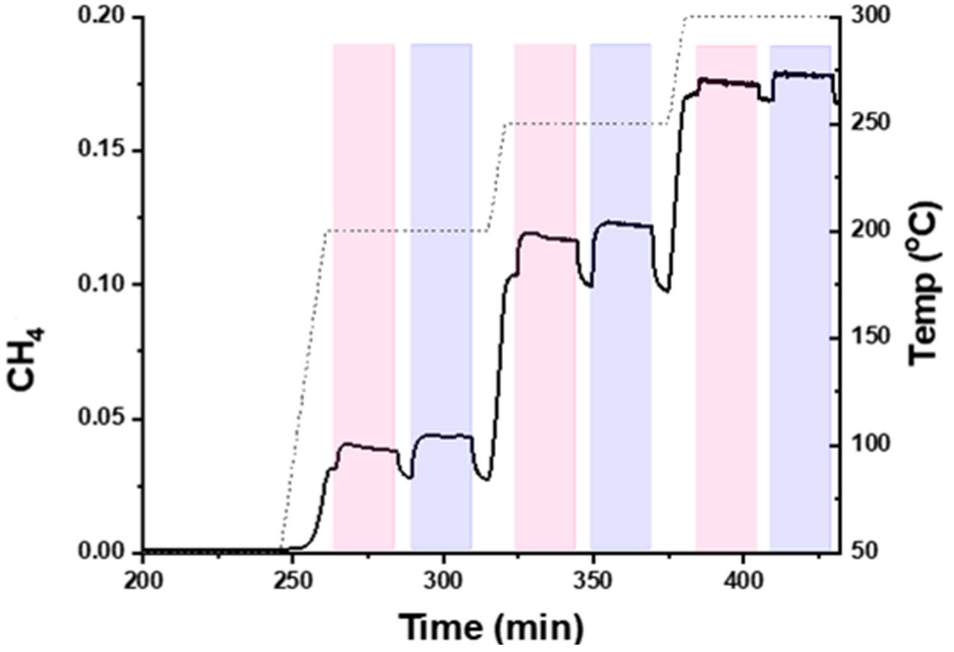

Figure. S6 Comparing the transient in CH_4 response of the mass spectrometer induced by visible light (pink), or UV/Vis (purple) illumination in the temperature range from 50°C to 300°C. See Fig. S1 for the emission lines which are filtered in the visible case. The CH_4 quantity is plotted in arbitrary units. The positive effect of illumination by UV radiation is relatively small on formation rate of CH_4.

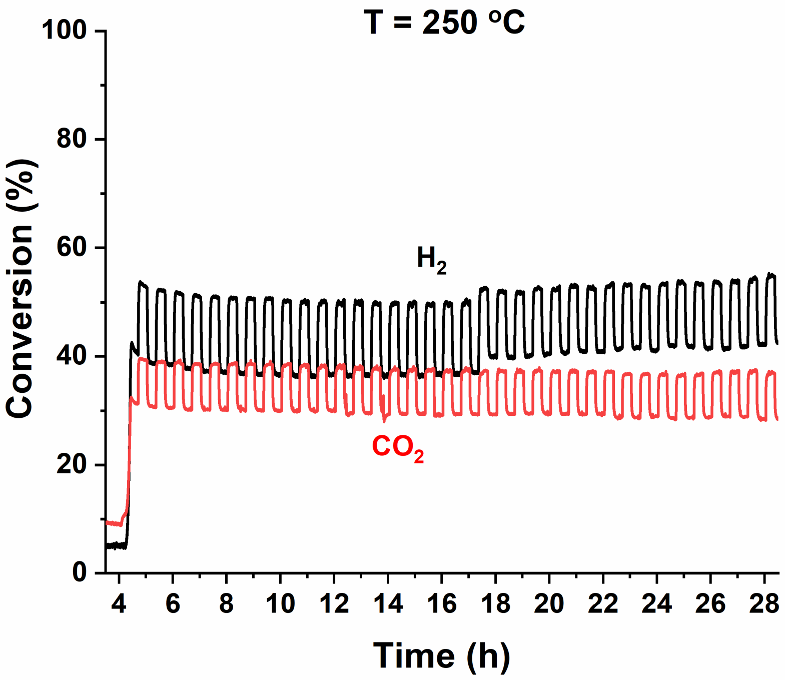

Figure. S7 Stability assessment of the Ru/TiO_2 using repetition of light-on, light-off cycles at 250 °C under chopped UV/Vis irradiation for 24h. Now the effect of light on the conversion of H_2 and CO_2 is shown - corresponding to the applied global temperature of 250 °C. The increase in conversion is again explicable by a temperature increase upon illumination of ~ 10 °C (to 260 °C).

Back-of-the-envelope calculation of the effect of illumination on temperature.

During UV/Vis illumination, heating of the sample might occur due to the energy of the light source, thus resulting in a thermal contribution to the catalysis. The intensity of the light source is 3.6 kW m^{-2} (360 $mW.cm^{-2}$) or 0.36 J s^{-1} cm^{-2}. The diameter of the sample holder cup is 0.59 cm, which corresponds to an area of 0.27 cm^2. This means that the energy input on the sample holder is 0.27 cm^2 * 0.36 J s^{-1} cm^{-2} = 0.098 J/s.

50 mg of Ru/TiO_2 was used. As TiO_2 is dominantly present, a molar heat capacity of 55.100 J mol^{-1} K^{-1} at 298 K (https://doi.org/10.2138/am.2009.3050) was used for calculations. Typically, the molar heat capacity $C_{P,m}^0$ in J mol^{-1} K^{-1} is calculated as follows:

$$C_{P,m}^0 = \frac{Q}{\Delta T * n} \quad \text{(S1)}$$

where Q is the energy input, ΔT is the change in temperature and n is the molar amount. Rewriting yields:

$$\Delta T = \frac{Q}{C_{P,m}^0 * n} \quad \text{(S2)}$$

Because:

$$n = \frac{m}{M} \quad \text{(S3)}$$

with m the mass (g) and M the molar mass (79.866 g/mol for TiO_2), equation 2 can be rewritten as follows:

$$\Delta T = \frac{Q * M}{C_{P,m}^0 * m} \quad \text{(S4)}$$

Using the given values yields the following maximum temperature increase per second can be calculated:

$$\frac{dT}{dt} = \frac{0.098 \text{ J s}^{-1} * 79.866\ g\ mol^{-1}}{55.100 \text{ J mol}^{-1} \text{ K}^{-1} * 0.050\ g} = 2.9\ K\ s^{-1}$$

Likely, the 'real' heating rate is lower than the calculated value, due to the following factors:

A 100% absorption is assumed. It is very likely that at least a part of the incident light is scattered to the environment, by for example the quartz window, and the sample powder.

Considering these losses, and given the fact that the productivity increase requires in the order of ~ 5 s (see the steep increase and decline of the shape of the light on – light off cycles in Figure S5 – the temperature increase of ~10 °C is in agreement with theory.

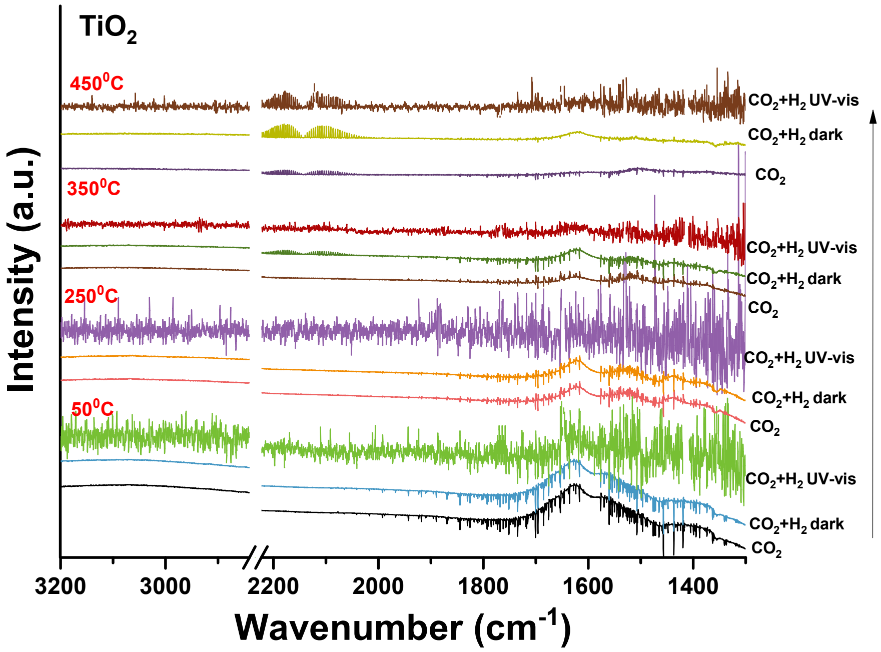

Figure. S8. In situ DRIFT spectra of photothermal hydrogenation of CO_2 on TiO_2 in the dark and under UV-vis irradiation; CH_4 is not observed – confirming that Ru is indispensable for converting CO_2 and H_2 into CH_4. The absorbance features at ~1580

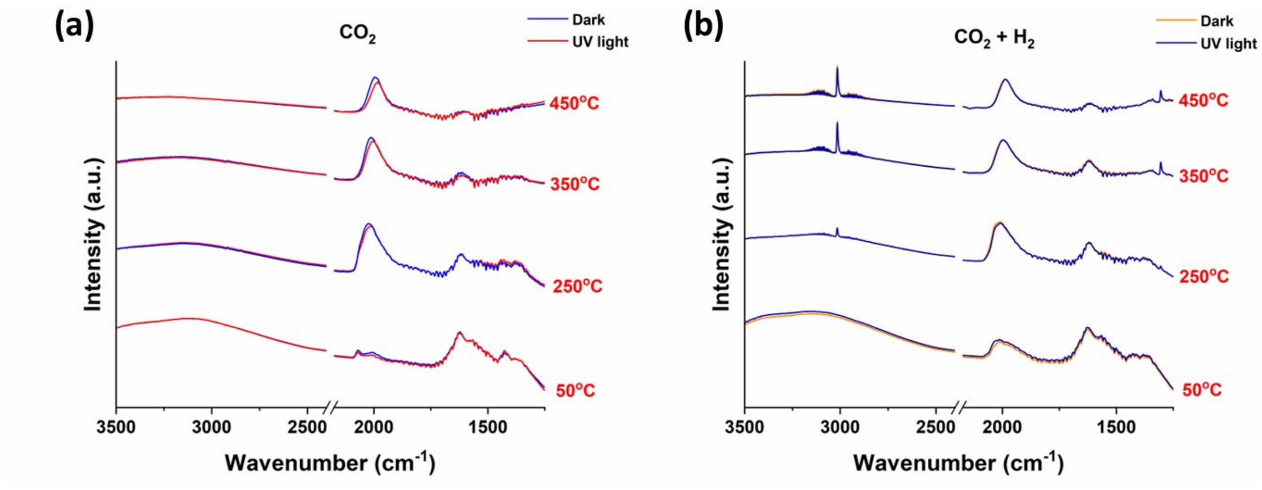

Figure. S9. **a:** In situ DRIFT spectra of Ru-CO formed by exposure of Hydrogen- reduced Ru/TiO_2 to CO_2 only (for 30 minutes). Spectra in the absence ('dark'- blue lines) and under UV irradiation ('UV-light – red lines) are compared at various temperatures. Please note that in particular at the highest temperature of 450 °C, a small difference in the intensity of the CO absorption band can be observed; **b:** In situ DRIFT spectra of Ru-CO formed by exposure of Hydrogen- reduced Ru/TiO_2 to CO_2 **ánd** Hydrogen (for 30 minutes in batch mode). UV LED illumination has no effect on the adsorbed CO intensity – nor on the amount of methane that is formed (see the rotational bands of gas phase methane at ~3000 cm^{-1}. Illumination was performed with a UV LED maximizing intensity at 370 nm – to the amount of ~ 7 mW/cm^2.

.

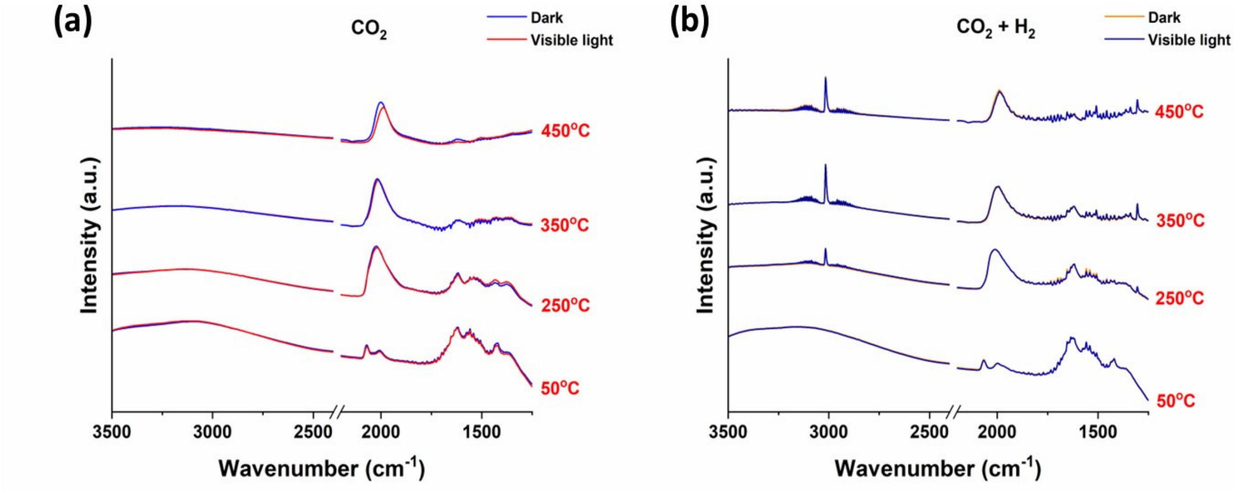

Figure. S10. **a:** In situ DRIFT spectra of Ru-CO formed by exposure of hydrogen- reduced Ru/TiO_2 to CO_2 only (for 30 minutes). Spectra in the absence ('dark'- blue lines) and under Visible irradiation (' Visible-light – red lines) are compared at various temperatures. Please note that in particular at the highest temperature of 450 °C, a small decrease in the intensity of the CO absorption band can be observed upon illumination; **b:** In situ DRIFT spectra of Ru-CO formed by exposure of hydrogen-reduced Ru/TiO_2 to CO_2 **and** hydrogen (for 30 minutes in batch mode). Now visible light illumination has no effect on the adsorbed CO intensity – nor on the amount of methane that is formed (see the rotational bands of gas phase methane at ~3000 cm^{-1}. Illumination was performed with a Visible light LED maximizing intensity at 530 nm (green light) – to the amount of ~ 8.5 mW/cm^2.
